# Risk–Benefit Assessment of an Increase in the Iodine Fortification Level of Foods in Denmark—A Pilot Study

**DOI:** 10.3390/foods11091281

**Published:** 2022-04-28

**Authors:** Lea Sletting Jakobsen, Josefine Ostenfeld Nielsen, Sophie Egesø Paulsen, Malene Outzen, Allan Linneberg, Line Tang Møllehave, Tue Christensen, Gitte Ravn-Haren

**Affiliations:** 1 National Food Institute, Technical University of Denmark, 2800 Kongens Lyngby, Denmark; s183487@student.dtu.dk (J.O.N.); s183452@student.dtu.dk (S.E.P.); maou@food.dtu.dk (M.O.); tuchr@food.dtu.dk (T.C.); girh@food.dtu.dk (G.R.-H.); 2 Center for Clinical Research and Prevention, Bispebjerg and Frederiksberg Hospital, 2000 Frederiksberg, Denmark; allan.linneberg@regionh.dk (A.L.); line.tang.moellehave@regionh.dk (L.T.M.); 3 Department of Clinical Medicine, Faculty of Health and Medical Sciences, University of Copenhagen, 2200 Copenhagen, Denmark

**Keywords:** iodine fortification, risk–benefit, public health impact, DALY

## Abstract

Iodine deficiency is one of the most common nutritional disorders worldwide. In Denmark, the mandatory iodine fortification of salt of 13 ppm was introduced in 2000 to eradicate mild to moderate iodine deficiency and the fortification level was increased to 20 ppm in 2019. However, the optimal iodine intake is a narrow interval, and the risk of disease increases with intakes both below and above this interval. In this study, we quantified the risk–benefit balance in the Danish adult population by increasing the mandatory fortification level. We applied a risk–benefit assessment approach in which population-level iodine intakes before and after the increase in fortification were integrated with epidemiological evidence of the association between iodine nutrition status and risk of relevant diseases to estimate the number of cases caused or prevented and estimated health impact in terms of disability-adjusted life years (DALY). We estimated an overall beneficial health impact and prevention of 34.9 (95% UI: −51.6; −21.7) DALY per 100,000 adults in the population annually with the increase in fortification level. Prevention of low IQ in children due to maternal iodine deficiency was the primary contributor to overall health gain. The gain in healthy life years comes at the expense of extra cases of goiter due to iodine excess. Due to lack of data, hypo- and hyperthyroidism related to iodine status were not included. Neither were children as a population group. Because of this, as well as uncertainties inherent in the model and data used, results should be interpreted with caution. We argue that nation-specific, quantitative assessments of the public health impact of fortification programs provide transparent, evidence-based decision support. Future research should aim to enable the inclusion of all relevant health effects as well as children in the assessment.

## 1. Introduction

Iodine deficiency (ID) and the associated adverse health effects is one of the most common nutritional disorders worldwide [1,2]. Iodine is essential for the synthesis of the thyroid hormones triiodothyronine (T3) and thyroxine (T4), which are involved in the regulation of numerous metabolic processes including normal brain development [3,4]. Iodine deficiency has been deemed the world’s greatest single cause of preventable brain damage [5] and fortification of salt and foods with iodine has been identified as an effective means to eradicate iodine deficiency and iodine deficiency disorders (IDD) [2]. In Denmark, mild-to-moderate iodine deficiency led to the implementation of mandatory iodine fortification of household salt and salt included as an ingredient in bread and bakery products in 2000 [6,7,8]. The effects of the Danish fortification program on iodine intake and thyroid disease patterns were thoroughly monitored in the following years by the Danish investigation on iodine intake and thyroid diseases (DanThyr) [9] as recommended by the World Health Organization (WHO) [2]. The introduction of iodine fortification led to increased iodine intakes in 2004–2005 and changes in biomarkers of thyroid function as well as incidence and type of thyroid diseases were observed in the general Danish population, including increases in the incidence of overt hypo- and hyperthyroidism [10,11]. However, a follow-up study performed in 2008–2010 revealed that the median urinary iodine concentration in women was below the recommendations [12]. These results, combined with data showing that Danish pregnant women are at risk of iodine deficiency [13,14], led the Danish food authorities to increase the iodine fortification level from 13 to 20 ppm in 2019, based on estimated iodine intakes in the general Danish population [8]. However, at this time no initiatives were taken to assess the consequences of the higher fortification level.

The recommended daily iodine intake (RI) is 150 µg for adults, and the iodine intake of a population should preferably be kept within a range (i.e., between the average requirement (AR) and upper tolerable intake level (UL) of 100 and 600 µg/day, respectively) to avoid adverse health effects associated with iodine deficiency or excessive intake [15,16]. Thus, it is essential to monitor and quantify the impact of iodine intake following the installment of a fortification program and changes thereof.

Risk–benefit assessment (RBA) is a tool that allows for quantitative comparison of human health risks and benefits from foods or food compounds based on a common scale of measurement [17,18]. The commonly used scale of measurement in RBA is the disability-adjusted life year (DALY). DALY is a composite health metric combining evidence of disease incidence, severity, and mortality. One DALY is equal to one year of healthy life lost, and enables the comparison of the impact on the health of various diseases and health outcomes [19,20]. In another study, RBA has been used to quantitatively evaluate the health impact in terms of DALY of a fortification program with folic acid in bread in the Netherlands, taking into account both the prevention of neural tube diseases and the increase in the risk of colorectal cancer among others [21]. RBA as a tool for setting adequate and safe micronutrient levels in foods was discussed by Bruins et al. (2015) [22].

In this pilot study, we quantified the health impact on the Danish population of increasing the mandatory iodine fortification level of household salt and salt included as an ingredient in bread and bakery products. We quantified the number of healthy life years gained or lost in the total and sub-groups of the Danish population due to the change in the mandatory fortification levels from 13 (previous fortification level) to 20 ppm (current fortification level). We also evaluated the impact of different assumptions on background iodine intake in two alternative scenarios.

## 2. Materials and Methods

### 2.1. Model Framework

The overall model framework applied to estimate the risk–benefit balance of changing the mandatory fortification level from 13 to 20 ppm consisted of four steps. All modeling is based on data obtained from previously published studies, and is described in detail in the following sections. First, we estimated the shift in the prevalence of the population with an iodine nutrition status interpreted as iodine deficient (ID) and with an iodine nutrition status interpreted as an iodine excessive state (IE) due to the change in fortification level. Then, the prevalence of exposure was integrated with epidemiological evidence of the association between ID and IE and the risk of relevant diseases to estimate the annual number of incident cases caused or prevented by the shift in the prevalence of exposure. Lastly, we attributed the difference in DALY between the fortification levels by applying data from health and population statistics. The output of the model was the DALY difference between scenarios of the estimated annual incident cases and the future burden ascribed to these cases. The uncertainty in model parameters was propagated by Monte Carlo simulations in a one-dimensional stochastic model (10,000 iterations). All models were developed in Microsoft Excel (vers. 2018) with the add-on software ModelRisk by Vose Software^TM^. All model parameters and distributional assumptions are presented in Tables S1–S4 in Supplementary Materials.

#### Definition of Iodine Deficiency and Iodine Excessive Intake

We defined levels for being in an ID or IE state based on recommendations for dietary reference values for iodine [15,16]. We assumed that individuals with an iodine intake below the AR of 100 µg/day are in a state of iodine deficiency. According to the Nordic Nutrition Recommendations (2012), pregnant women require an extra 25 µg/day of iodine [16]. Therefore, we assumed that pregnant women with an iodine intake below 125 µg/day are in a state of iodine deficiency. For the general adult population, we assumed that intakes above the UL of 600 µg g/day are excessive [15,16].

### 2.2. Selection of Health Effects

**Figure 1:**
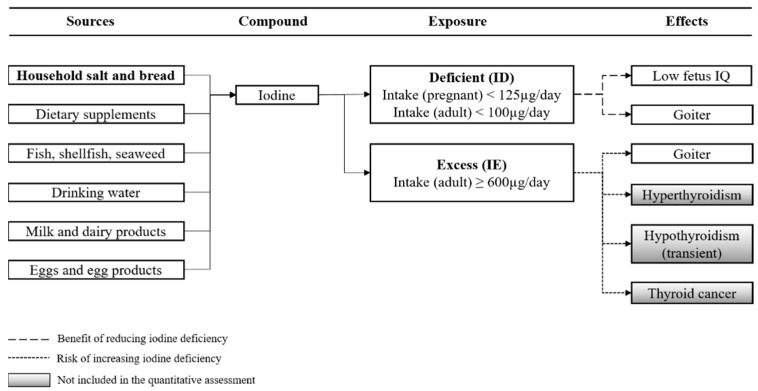
Conceptual model relating dietary sources of iodine to iodine nutrition status and related health effects. Bold text indicates iodine fortification. Beneficial effects are represented by reducing iodine deficiency and the prevention of health effects; adverse effects are represented by increasing excess iodine intake and causing health effects. Health effects in gray boxes are not included in the quantitative risk–benefit assessment.

We performed a literature search to identify health effects associated with ID and IE, respectively. A search string with keywords that link either ID or IE to adverse and/or beneficial health effects was run through the Library Discovery Tool of the Technical University of Denmark, DTU Findit (https://findit.dtu.dk/en/about/providers, accessed on 17 May 2021). The search was limited to papers in Danish and English, and relevant references in the literature identified in the primary search were also identified. For a health effect to be included in the quantitative risk–benefit assessment, we decided that there should be a convincing level of evidence of a causal relation between ID and IE and any health effects, and data available to estimate disease occurrence and DALY associated with the health effects. The evidence from the different studies was graded according to the criteria as proposed by the WHO. The strength of evidence was classified as either “convincing”, “probable”, “possible”, or “insufficient” [23]. Based on this, two health effects were selected for inclusion in our quantitative assessment, namely goiter (both associated with ID and IE) and low fetus intelligence quotient (IQ) (associated with ID) (Figure 1 and Table 1). For further descriptions of the included health effects see Appendix A. Hypo- and hyperthyroidism as well thyroid cancer (benign) are well-established health effects associated with excessive intakes of iodine. However, these health effects were not included because data to incorporate them quantitatively in the model was not readily available.

### 2.3. Prevalence of Exposure

The population distribution of the mean daily intake of iodine from all food sources and supplements was estimated based on data from the Danish National Survey of Dietary Habits and Physical Activity (DANSDA) conducted in 2011–13 [26], and thus represents the population iodine intake at the iodine fortification level of 13 ppm. The study population in DANSDA has been described in detail elsewhere but consists of a representative sample of 3946 individuals aged 4–75 years, selected randomly using the Danish Civil Registration System (CPR), and who had recorded all food and beverages consumed for 7 consecutive days [26]. The mean daily iodine intake in the general Danish population at a fortification level of 20 ppm was derived from Outzen et al. (2022) [27], in which simulations of DANSDA data to determine the expected influence of the fortification level of 20 ppm on iodine intakes were performed. The intake was in both the 13 and 20 ppm fortification levels estimated in µg/day for men and women in the age groups 18–75 and for women of childbearing age (15–49 years).

In order to estimate the prevalence of the population having an intake corresponding to either iodine deficiency or iodine excess, we defined distributions using the estimated iodine intakes (mean and associated standard deviations) of adult men and women (18–75 years) and women of childbearing age (15–49 years) at both fortification levels. It was assumed that the intake follows a lognormal distribution. The prevalence of exposure was estimated as the cumulative probability of belonging to a given category of iodine nutrition status; specifically, we estimated, for each fortification level, the prevalence of adult men and women having an intake <100 and >600 µg/day and of women of childbearing age having an intake <125 µg/day.

### 2.4. Estimating Changes in Incidence of Selected Health Outcomes

#### 2.4.1. Fetus IQ

To estimate the change in the incidence of children born with a low IQ due to the change in fortification level, we assumed a mean decrease in IQ points of 7.4 (95% uncertainty interval (UI): 6.9–10.2) in children of iodine-deficient mothers [24] (Table 1). This effect size is based on a meta-analysis including studies that have investigated the relationship between the iodine status of mothers during pregnancy and the IQ score of their children, as well as studies directly investigating the relationship between the iodine status of the children and their IQ score. The studies included in the meta-analysis are from all over the world and include children from 0–5 years of age. The population distribution of IQ scores is usually defined as normally distributed with a mean and standard deviation of 100 and 15, respectively, where an IQ below 85 is associated with intellectual disability [28]. We assumed this IQ distribution to represent the IQ distribution at a fortification level of 13 ppm. Using this information, we estimated the difference in probability of a child being born with an IQ < 85 between the 13 and 20 ppm fortification levels. Then, we estimated the additional annual incidence of children born with an IQ < 85 as the estimated number of births by deficient mothers using population statistics (Table S2).

#### 2.4.2. Goiter

We estimated the goiter incidence attributed to ID and IE for each fortification level by estimating the population attributable fraction (PAF), a measure of the fraction of total goiter incidence avoided when exposure to a risk factor is removed (i.e., ID and IE) by:

PAF = *p*_s,is_ (OR_is_ − 1)/(*p*_s,is_ (OR_is_ − 1) + 1) (1)

where *p*_s,is_ is the prevalence of exposure of iodine status (is) for either sex (s) and OR is the odds ratio for goiter depending on iodine status (either ID or IE) compared to an optimal iodine status. ORs for goiter in ID and IE were collected from Yu et al. (2008). The ORs are based on the association between iodine status and the prevalence of goiter in three cities in China with three different iodine intake levels: mildly deficient, more than adequate, and excessive. The study was an 11-year follow-up study and the cohort included 2708 men and women in the follow-up [25] (Table 1)

The DanThyr study monitored the goiter incidence following the first Danish mandatory fortification program (13 ppm), reporting an incidence rate of 16.6/1000 person years (py), but excluding the diffuse goiter [29]. In a German population with a similar iodine status as in Denmark under a mandatory fortification level of 13 ppm, an incidence rate of 34/1000 py was reported [30]. The German study also included diffuse goiter types, and we therefore assumed that this incidence rate was approximately the incidence rate in Denmark at a 13 ppm fortification level. We calculated the total goiter incidence based on the assumption that the total goiter incidence rate has a ratio of 4:1 between women and men [31]. The PAF was multiplied by the total goiter incidence to estimate the number of cases caused or prevented due to ID and IE at both the 13 and 20 ppm fortification levels (Table S3).

### 2.5. DALY and DALY Difference

The DALY for a given health outcome is calculated as the sum of years lived with disability (YLD) and the years of life lost due to premature mortality caused by the health outcome (YLL) [19]. YLD is the product of the incidence, duration, and severity of the health outcome. The severity is expressed by a disability weight (dw), which is a relative indicator on a scale from 0 to 1, where 0 is perfect health and 1 is death. YLL is the product of the mortality of the health outcome and the number of years lost to premature death, derived from WHO’s standard expected years of life lost [32].

DALY was estimated for each of the included health effects and summarized for each fortification level. The estimated overall health impact of the increase in fortification level from 13 to 20 ppm was estimated as:

Total DALY = ∑DALY_20 ppm_ − ∑DALY_13 ppm_, (2)

where DALY_20 ppm_ and DALY_13 ppm_ are the DALY for each of the included health effects and summed for each fortification level, respectively. All data used to estimate DALY are presented in Table 2.

To estimate the DALY of lower fetus IQ, we assumed that it is a lifelong condition, but that low IQ does not increase the risk of death. Therefore, only YLD contributes to fetus IQ. Disability weights were assigned to each class of intellectual disability. DALY caused by the goiter was estimated by assigning the proportion (4:1 between women and men) and duration of subtypes of goiter specific for men and women and the dw to the estimated goiter incidence. It was assumed that death is not caused by goiter itself and therefore only YLD contributed to overall DALY.

### 2.6. Scenario Analyses

We investigated the change in DALY in two alternative scenarios of background iodine intake from dietary sources and supplements. We investigated the impact of increasing the fortification level, if iodine intake data represented (scenario a) iodine intake data from food sources only, excluding iodine-containing supplements (i.e., representing a population where no one takes supplements), and (scenario b) iodine intake data from all food sources and the addition of 150 µg/day iodine (i.e., representing a population where all individuals use iodine-containing supplements of 150 µg/day).

## 3. Results

### 3.1. Iodine Intake and Prevalence of Exposure

Table 3 presents the iodine intake from foods including dietary supplements in the general male and female adult population and of women of childbearing age in both the 13 and 20 ppm fortification levels. Additionally, the iodine intake of children at both fortification levels is presented in Appendix B Table A1.

At the 13 ppm iodine fortification level, 1.5% and 4.2% of the adult men and women had an iodine intake below 100 µg/day (Table 4). With an increase in the fortification level, this prevalence decreased to 0.2% and 1.2%, respectively. Conversely, 0.4% and 0.1% of adult men and women, respectively, had an excessive iodine intake at the 20 ppm fortification level which is an increase from 0.2% and 0.07%, respectively, at the 13 ppm. The prevalence of women of childbearing age with an iodine intake below 125 µg/day decreased from 16% at the 13 ppm fortification level to 7% at 20 ppm (Table 4).

### 3.2. Incidence

The estimated annual number of new cases of low IQ and goiter at fortification levels of 13 and 20 ppm are presented in Table 5. The increased fortification level resulted in an approximately 55% decrease in the number of newborns with an IQ of 85 or below, due to the decrease in the prevalence of iodine-deficient mothers. The goiter incidence caused by ID was estimated to decrease from 8.5 to 1.4 cases per 100,000 adult men and from 94 to 28.4 cases per 100,000 adult women, corresponding to 54% and 70% decreases, respectively. However, the goiter incidence caused by IE was estimated to increase from 0.6 to 1.4 cases per 100,000 adult men and from 0.9 to 1.7 cases per 100,000 adult women.

### 3.3. Risk–Benefit Assessment in Terms of DALY

**Figure 2:**
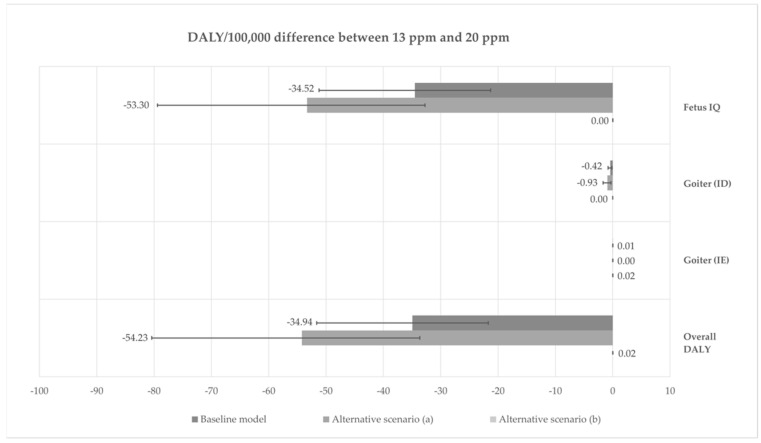
Annual DALY difference per 100,000 per health effect and summed over health effects between fortification levels 13 and 20 ppm for the baseline scenario and the two alternative scenarios.

The estimated decrease in the number of children born with a low IQ (below 85) due to maternal iodine deficiency corresponds with a decrease in DALY from 61.8 (95% UI: 38.1; 91.7) to 27.3 (95% UI: 16.8; 40.5) per 100,000 adults (Table 6). For an iodine deficiency-induced goiter, the decrease in the incidence between fortification levels corresponded with a decrease in DALY per 100,000 adults from 0.6 (95% UI: 0.2; 1.1) to 0.2 (95% UI: 0.1; 0.3) in women, and was negligible for men. The increase in DALY due to a goiter induced by excessive iodine intake was negligible for both men and women. The relatively low DALY estimated for goiter incidence is due to a low dw for goiter grades 1 and 2, a relatively short duration (between two and five years), and no associated mortality (Table 2). Increasing the fortification level from 13 to 20 ppm resulted in an overall annual DALY of –34.9 (95% UI: −51.6; −21.7) per 100,000 adults (baseline model, Figure 2). A low IQ was estimated to contribute to 34.5 of the DALY prevented by increasing the fortification level. Goiter cases prevented due to a lower prevalence of ID contributed to a 0.4 DALY prevented, whereas a goiter due to a higher prevalence of excessive iodine intakes caused less than 0.01 DALY per 100,000 adults.

### 3.4. Scenario Analysis

The proportion of men, women, and women of childbearing age being deficient or with an excessive intake at each fortification level for scenario (a) (iodine intake only from dietary sources) and scenario (b) (iodine intake from dietary sources and assuming all individuals in the population use iodine supplements with a daily content of 150 µg) are presented in Supplementary Materials Table S5–S10. The overall DALY results are for the two scenarios shown in Figure 2. In scenario (a), it was estimated that 54.2 DALY (95% UI: 33.7; 80.4) per 100,000 are prevented annually by the increase in fortification from 13 to 20 ppm, with the IQ of newborns being the main contributor to prevented DALY. In scenario b, increasing the fortification level resulted in 0.02 DALY (95% UI: 0.0; 0.04) per 100,000 adult population per year. Scenario (a) yields a slightly higher benefit from the increase in fortification than the baseline scenario. This is due to the fact that by assuming that only dietary sources are contributing to the iodine intake, the increase in fortification will prevent a higher proportion of the population from being iodine deficient. Conversely, as scenario (b) represents a population where everyone takes a standard supplement containing 150 µg iodine per day, only very few are iodine deficient at the 13 ppm fortification level, and thus the impact of iodine fortification is significantly smaller.

## 4. Discussion

We investigated the risk–benefit balance of increasing the iodine fortification level in Denmark from 13 to 20 ppm and estimated the potential population health impact of such a change accounting for health effects associated with inadequate and excessive intakes of iodine. We found that 34.5 DALY per 100,000 adult population would be prevented annually by increasing the fortification level to 20 ppm, corresponding to approximately 1600 healthy life years gained in Denmark per year. Our results show that the prevention of children born with a lower IQ due to maternal deficiency would be the main contributor to the overall health impact. Goiter contributed to a lesser extent compared with low IQ. Albeit changes in the goiter incidence associated with 13 and 20 ppm-level fortifications amounted to approximately 72 fewer cases per 100,000, the increase would result in less than one healthy life year saved per 100,000 adults.

As the impact of fortification programs depends on the baseline iodine status in a given population, we assessed the risk–benefit balance of hypothetical populations with iodine intakes from only dietary sources (scenario a) and populations in which all individuals take daily food supplements with 150 µg iodine in addition to dietary sources (scenario b). In the latter scenario, we found that the benefit of increasing the fortification level would no longer outweigh the adverse effects. Thus, under this scenario, the impact would be negligible. Our findings highlight the relevance of assessing the impact of fortification programs quantitatively and in composite health metrics to compare risks and benefits on a common scale.

Our model relied on a number of assumptions and had some limitations, and results should be interpreted with caution. We accounted for the selected health effects (low IQ of newborns due to deficient mothers and goiter as a result of both inadequate and excessive iodine intakes). Additionally, both hyper- and hypothyroidism as sequelae or independent of goiter, as well as benign thyroid cancer are established health outcomes of iodine excess [34], but were not included in our study. Those health effects can manifest in various ways, and interactions between goiter and hypo- and hyperthyroidism are complicated. While an increase in hyperthyroidism after the introduction of an iodine fortification program is well documented, the effect may be seen transiently in a population [35,36], and evidence as to how the magnitude of the increased iodine intake following fortification at 20 ppm affects the transient outcome is not readily available. The exclusion of hyper-, hypothyroidism, and benign thyroid cancer is likely to overestimate the beneficial effect of iodine fortification, but to an uncertain extent.

The odds ratios used to estimate the impact on the incidence of goiter is from a population with a higher median UIC compared with what is seen in the Danish population [29]. For the iodine-deficient region, the median UIC was close to what is considered AI by the WHO. This means that the OR linking goiter to ID most likely is underestimated when transferring the results to the Danish population. Thus, the beneficial effect of iodine fortification might be underestimated slightly.

Our estimates were performed for the adult Danish population. However, children are a population group of concern when implementing iodine fortification in populations where for example dairy products, which are important sources of iodine, are an integrated part of children’s diet. At a fortification level of 20 ppm, 5% of the boys aged 4–10 years and girls aged 4–6 years are estimated to exceed their respective tolerable upper intake levels established by EFSA (Table A1). Therefore, excluding children in the present risk–benefit assessment might result in an underestimation of the adverse impact of increasing the fortification level, but the magnitude of the impact is unknown. At the 13 ppm fortification level, 5% of girls aged 15–17 years have an estimated iodine intake below the AR; at the 20 ppm fortification level, the intake is increased, but still below the AR.

Our assessment was also hampered by data gaps or unquantified uncertainties in the model parameters. Particularly, we applied data on estimated dietary iodine intakes from the Danish National survey on Diet and Physical Activity [26] to determine the prevalence of exposure. However, urinary iodine concentration (UIC) would have provided more accurate estimates of the iodine status in the population. Likewise, both uncertainty and variability influence the conversion of UIC to dietary intake, but have not been taken into account in our assessment. As the health effects included in our assessment are associated with iodine nutrition status (ID or IE) and the levels defining being in either ID or IE state, the magnitude of the risk–benefit assessment relies on the accuracy of the intake estimates and UIC-dietary intake conversion.

Other studies have assessed the risks and benefits of iodine fortification programs [37,38,39], either semi-quantitatively or quantitatively using quality-adjusted life years. To our knowledge, this is the first study assessing the impact of iodine fortification in terms of DALY globally. Schaffner et al. (2021) [38] evaluated the impact of implementing a mandatory iodine fortification program compared to none in Germany. They included more health effects in their disease model compared with our study, but concluded that overall fortifying salt will have a beneficial health impact, despite also causing additional cases of hyperthyroidism. Generally, a comparison of estimates in health impact between studies is difficult, because baseline iodine status in different populations varies between studies. Rochau et al. (2020) [37] performed a literature review of decision-analytical modelling studies of the effect of iodine deficiency prevention strategies quantified in terms of QALY or life years lost/gained used for cost-effectiveness analysis. They concluded that few studies assess primary prevention strategies on the population level and that on the basis of identified studies, conclusions on health impact varies depending on research question and assumptions made. This highlights the need for national specific estimates of the health impact of fortification programs, such as the present. Furthermore, it could be relevant to include and evaluate the impact on other aspects such as direct and indirect costs [36]. Generating evidence to be able to update the present study including accounting for other relevant health effects and include the health impact associated with health effects observed in children exceeding the UL will be important for future studies.

## 5. Conclusions

Our findings support the increase in mandatory iodine fortification of salt and bakery products from 13 to 20 ppm, as we estimated approximately 1600 healthy life years annually are gained on the population level in Denmark. The gain in healthy life years comes at the expense of only very few extra cases of goiter. Our results should be interpreted with caution, as unquantified uncertainty around model parameters may impact overall DALY estimates, and some health effects sensitive to changes in iodine intakes were not accounted for, and children were not included in our assessment. Future research should aim to derive evidence to enable the inclusion of relevant health effects and children in the quantitative risk–benefit assessment. National specific, quantitative assessments of the public health impact of fortification programs provide transparent, evidence-based decision support.

## Figures and Tables

**Table 1:** Health effects associated with iodine deficiency (ID) and iodine excess (IE), graded level of evidence, target population, and identified effect sizes for each association.

Health Effect	Level of Evidence	Target Population	Dose-Response (95% CI)	Reference
Fetus IQ	Convincing	Women of childbearing age (15–49)	Average lower fetus IQ of 7.4 (6.9–10.2) IQ-points due to maternal ID.	[24]
Goiter	Convincing	Total adult population (18–75)	OR for mild ID = 1.83 (1.26; 2.65) OR for chronic IE = 1.46 (1.01; 2.11).	[25]

CI = confidence interval, IQ = intelligence quotient, OR = odds ratio.

**Table 2:** Parameters used to estimate DALY for each of the health outcomes included in the risk–benefit assessment.

Health Outcome	Disability Weight ^1^ [95% UI] ^2^	Duration (Years)
Fetal IQ	IQ (>85) = 0	81.5 ^3^
	IQ (70–85) = 0.011 [0.000; 0.020]	
	IQ (50–69) = 0.043 [0.026; 0.064]	
	IQ (35–49) = 0.100 [0.066; 0.142]	
	IQ (20–34) = 0.160 [0.107; 0.226]	
	IQ (<20) = 0.200 [0.133; 0.283]	
Goiter	Goiter grade 1 = 0.001	Men ^4^ = 2
	Goiter grade 2 = 0.025	Women ^4^ = 5

^1^ All disability weights collected from Salomon et al. (2015) [28]; ^2^ uncertainty propagated by assuming a PERT distribution with mean and 95% uncertainty intervals (UI) as the parameters most likely, minimum and maximum, respectively; ^3^ duration is the life expectancy of a newborn estimated as the life expectancy of newborn boys and girls weighted by the probability of a newborn being a boy or girl; ^4^ derived from Diez et al. (2005) [33].

**Table 3:** Dietary exposure of iodine in µg/day in the general Danish population at 13 and 20 ppm fortification levels simulated based on the Danish National Survey of Diet and Physical Activity, 2011–2013.

	Mean (SD)	5th Percentile	50th Percentile	95th Percentile
**13 ppm**				
Adult men (18–75 years)	230 (84)	118	220	363
Adult women (18–75 years)	200 (75)	102	191	328
Women of childbearing age (15–49)	198 (80)	102	184	339
**20 ppm ^1^**				
Adult men (18–75 years)	267 (90)	142	259	409
Adult women (18–75 years)	228 (80)	121	217	369
Women of childbearing age (15–49)	226 (84)	120	212	376

^1^ Intake levels at both fortification levels are derived from [27]. SD = standard deviation, ppm = parts per million.

**Table 4:** Prevalence of men and women (age 18–75) with an estimated iodine intake below 100 µg/day, defined as iodine deficiency (ID), or >600 µg/day, defined as iodine excess (IE), as well as the prevalence of women of childbearing age (age 15–49) with an insufficient intake (<125 µg/day) at 13 and 20 ppm fortification levels.

Target Population	Iodine Nutrition Status	Prevalence of Exposure at 13 ppm (%)	Prevalence of Exposure at 20 ppm (%)
Men (18–75)	ID (<100 µg/day)	1.47	0.23
	IE (>600 µg/day)	0.19	0.42
Women (18–75)	ID (<100 µg/day)	4.19	1.23
	IE (>600 µg/day)	0.07	0.13
Women of childbearing age (15–49)	ID (<125 µg/day)	16.15	7.13

**Table 5:** Annual mean incidence per 100.000 adult population for each health effect caused by iodine deficiency (ID) and iodine excess (IE) at 13 and 20 ppm fortification levels.

Health Outcome		Incidence/100,000 at 20 ppm [95% UI] ^1^	Incidence/100,000 at 20 ppm [95% UI] ^1^
Fetal IQ	IQ 70–85	25.3 [21.8; 30.3]	11.2 [9.6; 13.4]
	IQ 50–69	9.3 [7.5; 12.1]	4.1 [3.3; 5.4]
	IQ 35–49	0.4 [0.3; 0.6]	0.2 [0.1; 0.2]
	IQ 20–34	0.0 [0.0; 0.0]	0.0 [0.0; 0.0]
	IQ < 20	0.0 [0.0; 0.0]	0.0 [0.0; 0.0]
Goiter	ID (Men)	8.5 [2.5; 16.6]	1.4 [0.4; 2.7]
	ID (Women)	94.0 [27.8; 180.4]	28.4 [8.2; 55.5]
	IE (Men)	0.6 [0.0; 1.5]	1.4 [0.0; 3.2]
	IE (Women)	0.9 [0.0; 2.0]	1.7 [0.0; 4.0]

^1^ 95% uncertainty interval (UI) propagated from uncertainty in the effect sizes reported in Table 1.

**Table 6:** Annual DALY per 100,000 for each health effect at fortification levels of 13 and 20 ppm.

Health Outcome		DALY/100,000 at 20 ppm	DALY/100,000 at 20 ppm
Fetal IQ	IQ 70–85	24.8 [10.3; 42.7]	10.9 [4.6; 18.8]
	IQ 50–69	33.6 [18.5; 53.4]	14.8 [8.1; 23.6]
	IQ 35–49	3.2 [1.8; 5.2]	1.4 [0.8; 2.3]
	IQ 20–34	0.2 [0.1; 0.3]	0.1 [0.0; 0.1]
	IQ < 20	0.0 [0.0; 0.0]	0.0 [0.0; 0.0]
Goiter	ID (Men)	0.0 [0.0; 0.0]	0.0 [0.0; 0.0]
	ID (Women)	0.6 [0.2; 1.1]	0.2 [0.1; 0.3]
	IE (Men)	0.0 [0.0; 0.0]	0.0 [0.0; 0.0]
	IE (Women)	0.0 [0.0; 0.0]	0.0 [0.0; 0.0]

## Supplementary Materials

The following supporting information can be downloaded at: https://www.mdpi.com/article/10.3390/foods11091281/s1, Supplemental material S1: Tables S1–S4 Description of models and input parameters, Table S5: Alternative scenario (a): prevalence of iodine exposure, Table S6: Alternative scenario (b): Prevalence of iodine exposure, Table S7: Alternative scenario (a): Annual mean incidence per 100,000 for each health effect. Table S8: Alternative scenario (b): Annual mean incidence per 100.000 for each health effect, Table S9: Alternative scenario (a): Annual DALY per 100,000 for each health effect at fortification levels of 13 and 20 ppm, Table S10: Alternative scenario (b): Annual DALY per 100,000 for each health effect at fortification levels of 13 and 20 ppm. References [40,41,42,43,44] are cited in the supplementary materials.

## Appendix A

The included health effects are in the following described in more detail:

Fetus IQ: Fetal brain development begins a few weeks after conception and continues during the entire pregnancy, along with the first year after birth [34]. During pregnancy, the fetus is dependent on an adequate supply of maternal free T4, which is needed by the fetus to generate T3 for the thyroid-dependent neurodevelopment and for the promotion of growth hormone secretion. The consequences of inadequate supply depend upon the timing and severity of maternal iodine deficiency, cretinism being the most severe outcome, while subtle changes in cognitive and neurological function, such as decreased IQ, are seen in children of mothers with less severe ID [45]. Mild to moderate iodine deficiency during pregnancy has been linked to delayed mental development of the fetus, leading to an average lower IQ compared to fetuses of iodine sufficient women [24,46].

Goiter: Goiter, which is an enlarged thyroid gland, is defined as a total thyroid volume > 18 mL in women and > 25 mL in men, or by the presence of thyroid nodules (lumps) [47]. Depending on the size, goiter is classified as either grade 1 or 2 according to WHO. Goiter grade 1 is not visible, but diagnosed by palpation or by the presence of thyroid nodules found by ultrasound. Goiter grade 2 is identified as clearly visible swelling of the neck [47,48]. The development of goiter due to thyroid autonomous growth and function is a consequence of prolonged thyroid hyperactivity. This is caused by an insufficient iodine intake, forcing the thyroid gland to compensate to keep thyroid hormone levels within a normal range (euthyroidism). A possible mechanism might be the upregulation of H_2_O_2_, which may cause mutations of thyroid cells and lead to clusters of autonomous functioning follicular cells [34]. Excessive iodine intake is also associated with an increased risk of goiter. This is probably caused by reversible inhibition of thyroid function, known as the Wollf–Chaikoff effect. However, the mechanism is not as well-defined as for ID.

Goiter is divided into different subtypes. Goiter is initially diffuse (smooth enlargement) and non-toxic, which does not affect the thyroid hormone production (euthyroidism) [15]. Some goiters develop nodules and are classified as either uninodular or multinodular. Toxic nodular goiter, which overproduces thyroid hormones (hyperthyroidism), is a complication of nontoxic goiter. Toxic diffuse goiter, also called Graves’ disease, is dependent on genetics, but can be triggered by IE. Moreover, Hashimoto’s disease is an autoimmune disease, which also depends on genetics, but can be triggered by IE. It can occur with or without goiter and can be diffuse or nodular. It is nontoxic but can lead to decreased thyroid hormone production (hypothyroidism) [34].

## Appendix B

**Table A1:** Dietary exposure to iodine in µg/day in Danish children aged 4–17 years at 13 and 20 ppm fortification levels based on the Danish National Survey on Diet and Physical Activity, 2011–13. Numbers in bold indicate exposures below the (AR) and above the tolerable upper intake level (UL).

	AR ^1^	UL ^2^	Mean (sd)	5th Percentile	Median	95th Percentile
**13 ppm**						
Boys (4–6 years)	65	250	179 (42)	116	176	**253**
Boys (7–10 years)	65	300	191 (58)	102	185	278
Boys (11–14 years)	75	450	203 (60)	120	201	304
Boys (15–17 years)	100	500	220 (95)	103	201	380
Girls (4–6 years)	65	250	161 (45)	90	160	228
Girls (7–10 years)	65	300	177 (53)	105	172	255
Girls (11–14 years)	75	450	162 (61)	88	153	253
Girls (15–17 years)	100	500	168 (80)	**77**	169	300
**20 ppm**						
Boys (4–6 years)	65	250	204 (45)	134	203	**277**
Boys (7–10 years)	65	300	220 (64)	124	217	**321**
Boys (11–14 years)	75	450	237 (65)	144	233	344
Boys (15–17 years)	100	500	259 (104)	118	239	455
Girls (4–6 years)	65	250	184 (47)	111	183	**259**
Girls (7–10 years)	65	300	204 (55)	128	196	286
Girls (11–14 years)	75	450	190 (66)	111	184	293
Girls (15–17 years)	100	500	194 (84)	**97**	195	325

^1^ AR values for age-groups from 15 years and above established by [16], AR values for children below 15 years established by [49]. ^2^ UL for all age groups established by EFSA [15].
