# Supplementary material for: Risk–Benefit Assessment of an Increase in the Iodine Fortification Level of Foods in Denmark—A Pilot Study
Source: Foods. 2022 Apr 28;11(9):1281. doi: 10.3390/foods11091281 (PMC9104615; doi:10.3390/foods11091281)
Supplement: Supplementary file 1 [file foods-11-01281-s001.zip › Supplemental Tables S1_S2_S3_S4.pdf]

Supplemental Material S1

Table S1 – Exposure assessment input, equations, and distribution assumptions. Performed for both the previous fortification level of 13 ppm and the current fortification level of 20 ppm.

Input/Equation/Distribution	Description
$IN_{s,sc} = Lognormal(Mean_{s,sc}, Sd_{s,sc})$	Distribution: Intake distributions computed for each sex (s) - adult men (m) and women (w) and women of childbearing age (wc) - within each scenario (sc) - baseline, a and b.
$PE_{ID,s,sc} = P(IN_{s,sc} < 100) = F(100)$<br>$PE_{IE,s,sc} = P(IN_{s,sc} \geq 600) = 1 - F(600)$	Output: The prevalence of exposure (PE) of an iodine intake <100 or ≥600 µg/day (ID and IE), calculated for each sex (s), men and women, as cumulative probabilities for each scenario (sc).
$PE_{ID,wc,sc} = P(IN_{wc,sc} < 125) = F(125)$	Output: The prevalence of exposure (PE) of women of childbearing age (wc) with an insufficient iodine intake <125 µg/day (ID), calculated as cumulative probabilities for each scenario (sc).

Table S2 – Fetus IQ model input, equations, distribution assumptions. Performed for both the previous fortification level of 13 ppm and the current fortification level of 20 ppm.

Input/Equation/Distribution	Description
$B = 63,461$	Input: Number of annual births. Statistikbanken (folketal 4. kvartal 2021).
$B_{ID,sc} = B \cdot PE_{ID,wc,sc}$	Output: Number of annual births of iodine deficient (ID) women of childbearing age (wc), estimated for each scenario (sc).
$IQ_{ST} = Normal\,(100, 15)$	Distribution: The standard IQ distribution computed as a normal distribution with a mean 100 and a standard deviation 15 [1]. Describes variability in IQ.
$X_{IQ} = Triangle\,(89.8, 92.6, 93.1)$	Distribution: The mean of the IQ distribution for children born of deficient women, 100-7.4 (95% CI: 100-10.2; 100-6.9) [2]. Describes uncertainty of the effect of ID on IQ.
$IQ_{ID} = Normal\,(X_{IQ}, 15)$	Distribution: The IQ distribution of children born of deficient women computed as a normal distribution with mean X_{IQ} and standard deviation 15. Describes variability in IQ, with uncertainty linked to the mean of the distribution.
$P\,(70 \leq IQ \leq 85) = F(85) - F(70)$<br>$P\,(50 \leq IQ \leq 69) = F(69) - F(50)$<br>$P\,(35 \leq IQ \leq 49) = F(49) - F(35)$<br>$P\,(20 \leq IQ \leq 34) = F(34) - F(20)$<br>$P\,(\mathrm{IQ} \leq 20) = F(20)$	Output: The cumulative probability of the fetus being born with an IQ score within the 5 intervals below 85, associated with disability. Computed as point estimates for both the standard (ST) and deficient (ID) IQ distribution.

Continued on next page

Input/Equation/Distribution	Description
$P_{IQ(70-85)} = P(70 \leq IQ_{ID} \leq 85) - P(70 \leq IQ_{ST} \leq 85)$<br>$P_{IQ(50-69)} = P(50 \leq IQ_{ID} \leq 69) - P(50 \leq IQ_{ST} \leq 69)$<br>$P_{IQ(35-49)} = P(35 \leq IQ_{ID} \leq 49) - P(35 \leq IQ_{ST} \leq 49)$<br>$P_{IQ(20-34)} = P(20 \leq IQ_{ID} \leq 34) - P(20 \leq IQ_{ST} \leq 34)$<br>$P_{IQ(\leq 20)} = P(IQ_{ID} \leq 20) - P(IQ_{ST} \leq 20)$	Output: The additional probability of children being born with an IQ within the intervals below 85, due to maternal ID, compared to the standard probability when being born of women with optimal iodine intake.
$I_{IQ,sc} = B_{ID,sc} \cdot P_{IQ}$	Output: The additional annual incidence of children of deficient women being born with an IQ within the 5 intervals below 85. Calculated for each IQ interval and for each scenario (sc).
$N_{DK} = 4,683,525$	Input: Point estimates of the number of adults in Denmark. Statistikbanken (folketal 1. kvartal 2021) [3].
$IR_{IQ,sc} = \frac{I_{IQ,sc}}{N_{DK}} \cdot 100,000$	Output: Conversion of the annual incidence (cases/year) to incidence rate (cases/100,000 adult in DK).
$DW_{IQ(70-85)} = Pert\,(0.005, 0.011, 0.020)$<br>$DW_{IQ(50-69)} = Pert\,(0.026, 0.043, 0.064)$<br>$DW_{IQ(35-49)} = Pert\,(0.066, 0.100, 0.142)$<br>$DW_{IQ(20-34)} = Pert\,(0.107, 0.160, 0.226)$<br>$DW_{IQ(\leq 20)} = Pert\,(0.133, 0.200, 0.283)$	Distribution: The disability weights linked to the five IQ intervals below 85 points. Computed as Pert distributions with the mean and the 95% CI [4]. Describes uncertainty.
$P_{Boy} = 51.16\%$<br>$P_{Girl} = 48.84\%$	Input: Point estimates of the probability of giving birth to a boy or a girl from Statistikbanken 2020 [3].
$LE_{Men} = 79.5$<br>$LE_{Women} = 83.6$	Input: Point estimates of the life expectancy (no. years) at birth for men and women in Denmark from Statistikbanken 2019:2020 [3].
$LE_{Newborn} = (LE_{Men} \cdot P_{Boy}) + (LE_{Women} \cdot P_{Girl})$	Output: Point estimate of the weighted average life expectancy of a newborn at birth.
$YLD_{IQ,sc} = I_{IQ,sc} \cdot DW_{IQ} \cdot LE_{Newborn}$	Output: Estimated years lived with disability of low IQ caused by ID in Denmark. Calculated for each IQ interval, for each scenario (sc).
$DALY_{sc} = \sum(YLD_{IQ,sc})$	Output: Estimated total disability adjusted life years, calculated as the sum of the YLDs for each IQ interval. Estimated for each scenario (sc).
$DALY_{sc}\,rate = \frac{DALY_{sc}}{N_{DK}} \cdot 100,000$	Output: Conversion of DALY to rates per 100,000 adult in DK, for each scenario (sc).

Table S3 – Goiter model input, equations, and distribution assumptions. Performed for both the previous fortification level of 13 ppm and the current fortification level of 20 ppm.

Input/Equation/Distribution	Description
$OR_{ID} = Lognormal\,(1.26, 1.83, 2.65)$<br>$OR_{IE} = Lognormal\,(1.01, 1.46, 2.11)$	Distribution: The odds ratios linked to the risk of developing goiter due to both iodine status (is) - ID and IE [5]. Computed as log-normal distributions.
$PAF_{is,s,sc} = \frac{PE_{is,s,sc} \cdot (OR_{is} - 1)}{PE_{is,s,sc} \cdot (OR_{is} - 1) + 1} \cdot 100$	Output: The population attributable fraction. Calculated for both iodine status (is) for each sex (s), within each scenario (sc).
$IR = Pert\,(30.7, 34, 37.7)$	Distribution: The total goiter incidence rate per 1000 py, assumed to simulate the Danish adult population [6]. Computed as a pert distribution.
$I_{DK} = \frac{IR}{1000} \cdot N_{DK}$<br>$I_M = \frac{I_{DK}}{5}$<br>$I_W = \frac{I_{DK}}{5} \cdot 4$	Output: The estimated total annual goiter incidence, along with the total annual incidence for each sex (s) - men and women in Denmark - based on the the ratio of the incidence of 4:1 for women and men respectively [7].
$I_{is,s,sc} = I_s \cdot PAF_{is,s,sc}$	Output: The estimated total annual goiter incidence caused by either iodine status (is) for each sex (s), within each scenario (sc).
$IR_{is,s,sc} = \frac{I_{is,s,sc}}{N_{DK}} \cdot 100,000$	Output: Conversion of annual incidence (cases/year) to incidence rate (cases/100,000 adults).
$I,G2_{is,s,sc} = I_{is,s,sc} \cdot 0.01$<br>$I,G1_{is,s,sc} = I_{is,s,sc} - I,G2_{is,s,sc}$	Output: The incidence of goiter grade 1 and 2, wherer goiter grade 2 has a prevalence of $\approx 1\%$. Estimated for each iodine status (is) and sex (s), within each scenario (sc) [8].
$DW,G1 = 0.001$<br>$DW,G2 = 0.025$	Input: Point estimates of the disability weights associated with goiter grade 1 and grade 2 [9].
$D_M = 2$<br>$D_W = 5$	Input: Point estimates of the gender-specific duration of goiter. The variation is integrated in the model as the mean duration: Men duration: 2 years [0.5; 6], Women duration: 5 years [2; 11.8] [10].
$YLD_{is,s,sc} = (I,G1_{is,s,sc} \cdot DW,G1 \cdot D_s)$<br>$+ (I,G2_{is,s,sc} \cdot DW,G2 \cdot D_s)$	Output: The estimated years lived with disability due to goiter caused by exposure either iodine satus (is), for each sex (s), within each scenario (sc).
$DALY_{is,sc} = YLD_{is,m,sc} + YLD_{is,w,sc}$	Output: The estimated disability adjusted life years, estimated as the sum of the YLD for each sex - men (m) and women (w) for each iodine status (is) within each scenario (sc).
$DALY_{is,sc}\,rate = \frac{DALY_{is,sc}}{N_{DK}} \cdot 100,000$	Output: Conversion of YLD and DALY to rates per 100,000 adult in DK for each iodine status (is) within each scenario (sc).

Table S4 – Overall health impact equations.

Equation	Description
$\Delta DALY_{sc}\,rate = DALY_{FL20,sc}\,rate - DALY_{FL13,sc}\,rate$	Output: The change in DALY rate between the current fortification level (20 ppm) and the previous fortification level (13 ppm). Calculated for each health effect (IQ caused by maternal ID, goiter caused by ID, and goiter caused by IE) within each scenario (sc).
$net\,DALY\,rate = \sum(\Delta DALY_{sc}\,rate)$	Output: The overall change in DALY rate for all health effects, between the previous and current fortificaiton level. Calculated for each scenario (sc).

References

[1] Mensa Danmark. *Mensa - ofte stillede spørgsmål*. 2015. URL: http://mensa.dk/om-mensa/faq.

[2] K. Bougma et al. “Iodine and mental development of children 5 years old and under: A systematic review and meta-analysis”. In: *Nutrients* 5.4 (2013), pp. 1387–1416. ISSN: 20726643. DOI: 10.3390/nu5041384.

[3] Danmarks Statistik. *Statistikbanken - Befolkning og Valg*. 2020. URL: https://www.statistikbanken.dk/statbank5a/default.asp?w=1440.

[4] J. A. Salomon et al. “Disability weights for the Global Burden of Disease 2013 study”. In: *The Lancet Global Health* 3.11 (2015), e712–e723. ISSN: 2214109X. DOI: 10.1016/S2214-109X(15)00069-8.

[5] X. Yu et al. “A five-year follow-up study of goiter and thyroid nodules in three regions with different iodine intakes in China”. In: *Journal of Endocrinological Investigation* 31.3 (2008), pp. 243–250. ISSN: 03914097. DOI: 10.1007/BF03345597.

[6] H. Völzke et al. “Five-year change in morphological and functional alterations of the thyroid gland: The study of health in pomerania”. In: *Thyroid* 22.7 (2012), pp. 737–746. ISSN: 10507256. DOI: 10.1089/thy.2011.0525.

[7] Medscape. *How does the incidence of goiter vary by sex*. 2020. URL: https://www.medscape.com/answers/120034-102152/how-does-the-incidence-of-goiter-vary-by-sex.

[8] N. Knudsen et al. “Goitre prevalence and thyroid abnormalities at ultrasonography: A comparative epidemiological study in two regions with slightly different iodine status”. In: *Clinical Endocrinology* 53.4 (2000), pp. 479–485. ISSN: 03000664. DOI: 10.1046/j.1365-2265.2000.01121.x.

[9] WHO. *Age-specific disability weights for untreated and treated forms of sequelae included in the Global Burden of Disease Study*.

[10] J. J. Díez. “Goiter in adult patients aged 55 years and older: Etiology and clinical features in 634 patients”. In: *Journals of Gerontology - Series A Biological Sciences and Medical Sciences* 60.7 (2005), pp. 920–923. ISSN: 10795006. DOI: 10.1093/gerona/60.7.920.
