# Supplementary material for: Risk–Benefit Assessment of an Increase in the Iodine Fortification Level of Foods in Denmark—A Pilot Study
Source: Foods. 2022 Apr 28;11(9):1281. doi: 10.3390/foods11091281 (PMC9104615; doi:10.3390/foods11091281)
Supplement: Supplementary file 1 [file foods-11-01281-s001.zip › Supplementary tables_S5_S10.pdf]

Table S5. Alternative scenario a: prevalence of men and women (age 18-75) with an estimated iodine intake below 100 µg/day, defined as iodine deficiency (ID), or > 600 µg/day, defined as iodine excess (IE), as well as the prevalence of women of childbearing age (age 15-49) with an insufficient intake (< 125 µg/day) at 13 ppm and 20 ppm fortification levels.

**Target population**	**Iodine nutrition status**	**Prevalence of exposure at 13 ppm (%)**	**Prevalence of exposure at 20 ppm (%)**
Men (18-75)	ID (<100 µg/day)	3.06	0.55
	IE (>600 µg/day)	0.07	0.18
Women (18-75)	ID (<100 µg/day)	10.15	3.12
	IE (>600 µg/day)	0.01	0.02
Women of childbearing age (15-49)	ID (<125 µg/day)	26.10	12.06

Table S6. Alternative scenario b: Prevalence of men and women (age 18-75) with an estimated iodine intake below 100 µg/day, defined as iodine deficiency (ID), or > 600 µg/day, defined as iodine excess (IE), as well as the prevalence of women of childbearing age (age 15-49) with an insufficient intake (< 125 µg/day) at 13 ppm and 20 ppm fortification levels.

**Target population**	**Iodine nutrition status**	**Prevalence of exposure at 13 ppm (%)**	**Prevalence of exposure at 20 ppm (%)**
Men (18-75)	ID (<100 µg/day)	0.00	0.00
	IE (>600 µg/day)	0.45	1.57
Women (18-75)	ID (<100 µg/day)	0.00	0.00
	IE (>600 µg/day)	0.04	0.13
Women of childbearing age (15-49)	ID (<125 µg/day)	0.00	0.00

Table S7. Alternative scenario a: Annual mean incidence per 100.000 for each health effect caused by iodine deficiency (ID) and iodine excess (IE) at 13 ppm and 20 ppm fortification levels.

**Health outcome**		**Incidence/100.000 at 13 ppm [95% UI][1)]**	**Incidence/100.000 at 20 ppm [95% UI][1)]**
Fetal IQ	IQ 70-85	40.9 [35.4; 49.0]	18.9 [16.3; 22.6]
	IQ 50-69	15.0 [12.2; 19.6]	7.0 [5.6; 9.1]
	IQ 35-49	0.6 [0.5; 0.9]	0.3 [0.2; 0.4]
	IQ 20-34	0.0 [0.0; 0.0]	0.0 [0.0; 0.0]
	IQ < 20	0.0 [0.0; 0.0]	0.0 [0.0; 0.0]
Goitre	ID (Men)	17.4 [5.4; 32.9]	3.2 [1.0; 6.2]
	ID (Women)	215.8 [69.7; 393.4]	70.9 [21.8; 134.1]
	IE (Men)	0.2 [0.0; 0.5]	0.6 [0.0; 1.36]
	IE (Women)	0.1 [0.0; 0.3]	0.2 [0.0; 0.5]

[1)] 95% uncertainty interval propagated from uncertainty in the dose response relationships (table 1).

Table S8. Alternative scenario b: Annual mean incidence per 100.000 for each health effect caused by iodine deficiency (ID) and iodine excess (IE) at 13 ppm and 20 ppm fortification levels.

**Health outcome**		**Incidence/100.000 at 13 ppm [95% UI][1)]**	**Incidence/100.000 at 20 ppm [95% UI][1)]**
Fetal IQ	IQ 70-85	0.0 [0.0; 0.0]	0.0 [0.0; 0.0]
	IQ 50-69	0.0 [0.0; 0.0]	0.0 [0.0; 0.0]
	IQ 35-49	0.0 [0.0; 0.0]	0.0 [0.0; 0.0]
	IQ 20-34	0.0 [0.0; 0.0]	0.0 [0.0; 0.0]
	IQ < 20	0.0 [0.0; 0.0]	0.0 [0.0; 0.0]
Goitre	ID (Men)	0.0 [0.0; 0.0]	0.0 [0.0; 0.0]
	ID (Women)	0.0 [0.0; 0.0]	0.0 [0.0; 0.0]
	IE (Men)	1.5 [0.0; 3.4]	5.1 [0.1; 11.6]
	IE (Women)	0.5 [0.0; 1.0]	0.2 [0.0; 0.5]

[1)] 95% uncertainty interval propagated from uncertainty in the dose response relationships (table 1).

Table S9. Alternative scenario a: Annual DALY per 100.000 for each health effect at fortification levels of 13 ppm and 20 ppm.

Health outcome		DALY/100.000 at 13 ppm	DALY/100.000 at 20 ppm
Fetal IQ	IQ 70-85	39.5 [16.0; 68.9]	18.3 [7.4; 31.9]
	IQ 50-69	54.1 [29.8; 86.1]	25.0 [13.8; 39.8]
	IQ 35-49	5.2 [3.0; 8.4]	2.4 [1.4; 3.9]
	IQ 20-34	0.3 [0.1; 0.4]	0.1 [0.1; 0.2]
	IQ < 20	0.0 [0.0; 0.0]	0.0 [0.0; 0.0]
Goitre	ID (Men)	0.0 [0.0; 0.1]	0.0 [0.0; 0.0]
	ID (Women)	1.3 [0.4; 2.4]	0.4 [0.1; 0.8]
	IE (Men)	0.0 [0.0; 0.0]	0.0 [0.0; 0.0]
	IE (Women)	0.0 [0.0; 0.0]	0.0 [0.0; 0.0]

Table S10. Alternative scenario b: Annual DALY per 100.000 for each health effect at fortification levels of 13 ppm and 20 ppm.

Health outcome		DALY/100.000 at 13 ppm	DALY/100.000 at 20 ppm
Fetal IQ	IQ 70-85	0.0 [0.0; 0.0]	0.0 [0.0; 0.0]
	IQ 50-69	0.0 [0.0; 0.0]	0.0 [0.0; 0.0]
	IQ 35-49	0.0 [0.0; 0.0]	0.0 [0.0; 0.0]
	IQ 20-34	0.0 [0.0; 0.0]	0.0 [0.0; 0.0]
	IQ < 20	0.0 [0.0; 0.0]	0.0 [0.0; 0.0]
Goitre	ID (Men)	0.0 [0.0; 0.0]	0.0 [0.0; 0.0]
	ID (Women)	0.0 [0.0; 0.0]	0.0 [0.0; 0.0]
	IE (Men)	0.0 [0.0; 0.0]	0.0 [0.0; 0.0]
	IE (Women)	0.0 [0.0; 0.0]	0.0 [0.0; 0.0]
